# Ovarian Vein Thrombosis: A Sequela of COVID-Associated Coagulopathy

**DOI:** 10.7759/cureus.36437

**Published:** 2023-03-20

**Authors:** Shlok V Patel, Stuti Shah, Rina Patel, Shriya Bavishi, Yashvi Pethani, Kalp Shah

**Affiliations:** 1 Orthopedics, Byramjee Jeejabhoy (BJ) Medical College, Ahmedabad, IND; 2 Internal Medicine, Byramjee Jeejabhoy (BJ) Medical College, Ahmedabad, IND; 3 Obstetrics and Gynecology, Sardar Vallabhbhai Patel Hospital, Ahmedabad, IND; 4 Internal Medicine, Nathiba Hargovandas Lakhmichand (NHL) Municipal Medical College, Ahmedabad, IND

**Keywords:** coronavirus disease 19, venous thromboembolism, ovarian vein thrombosis, hypercoagulability, covid-19

## Abstract

Coronavirus disease 2019 (COVID-19) causes endothelial damage, blood stasis, and an overall state of hypercoagulability. This makes COVID a huge risk factor for venous thromboembolism (VTE) and arterial thromboembolism (ATE). Twenty percent of COVID-19 patients suffer from coagulation abnormalities like pulmonary embolism, myocardial infarction, stroke, deep vein thrombosis, etc. Ovarian vein thrombosis (OVT) has been previously linked to post-partum period, pregnancy, hypercoagulable state, or malignancy. We analyzed PubMed and Google Scholar databases for research and publications regarding OVT in patients with COVID-19. The search yielded nine case reports. These case reports were found to implicate COVID-associated coagulopathy (CAC) as an additional risk factor for ovarian vein thrombosis (OVT). OVT most commonly presents with abdominal pain and fever, making it difficult to diagnose, owing to the similarity in presentation with multiple other pathologies. OVT can be diagnosed radiologically with ultrasound, magnetic resonance imaging (MRI) scan, or CT scan with IV contrast. CT has been used as the modality of choice for diagnosing OVT. Although rare, OVT can cause life-endangering complications by extension of thrombus into systemic veins or pulmonary artery embolization. Therefore, early diagnosis and treatment are vital. There is no official guideline for the treatment of OVT post-COVID. However, the literature supports the use of apixaban or enoxaparin/acenocoumarol.

## Introduction and background

Coronavirus disease 2019 (COVID-19) is an infectious disease caused by severe acute respiratory syndrome coronavirus-2 (SARS-CoV-2). COVID-19 has infected millions of people and given rise to a global pandemic in just a few months [1,2]. The disease course of COVID-19 infection has been divided into four stages as follows: (1) upper respiratory tract infection, (2) onset of pneumonia, (3) hyperinflammatory state (characterized by cytokine storm leading to a deteriorating clinical condition), and (4) death or recovery [3,4].

A total of 20% of COVID-19 patients suffer from coagulation abnormalities. Such abnormalities mostly occur in severe and critical COVID-19 patients, i.e., in the third stage [2,3]. A study of 4906 hospitalized COVID-19 patients conducted by Giannis et al. in 2021 reported a 1.55% incidence rate of venous thromboembolism (VTE) post-discharge [5].

Multiple factors have been considered responsible for the hypercoagulability linked with COVID-19, also known as COVID-19-associated coagulopathy (CAC) [6]. Virchow’s triad describes three following causes of thromboses: intravascular vessel wall damage, stasis of blood flow, and hypercoagulable state [7,8]. COVID-19 involves the whole triad in its pathogenesis. Endothelial cells have a large number of angiotensin-converting enzyme 2 (ACE-2) receptors, which allow SARS-CoV-2 to enter the cells. The resulting direct invasion of endothelial cells, coupled with an increase in cytokines, acute phase reactants, and prothrombotic factors like von Willebrand factor (vWF), factor VIII, D-dimer, fibrinogen, etc. causes endothelial injury and subsequent thrombophilia [6,8].

Pulmonary embolism (PE) and deep vein thromboses (DVT) of limbs are accountable for the majority of VTE cases in the setting of coronavirus disease 2019 infection. Ovarian vein thrombosis (OVT), as a consequence of COVID-associated coagulopathy, is an unusual but very serious condition. It occurs most commonly after childbirth in around 0.05-0.018% of vaginal deliveries, and up to 2% of cesarean sections [9,10]. Other rare causes of OVT include malignancy, abdominal/pelvic surgeries, sepsis, and pelvic inflammatory disease [9,11]. The most common symptom of OVT is pain in the ipsilateral lower abdominal quadrant as that of the thrombosed vein. The pain can radiate to the upper abdomen, flank, or groin. Abdominal pain is most commonly accompanied by fever. The most specific feature of OVT is a palpable abdominal mass (46% of cases). Other non-specific symptoms include nausea, vomiting, anorexia, malaise, and ileus [12].

Early diagnosis and treatment of OVT are crucial. If untreated, it can cause life-threatening complications via thrombus extension into systemic veins and ensuing pulmonary embolization [13]. Sepsis, ovarian infarction, ovarian abscess, septic thrombophlebitis, uterine necrosis, and ureteral compression are some of the other critical complications [11]. Because the symptoms are vague and may mimic an acute abdomen, a high index of clinical suspicion integrated with the employment of imaging modalities, such as Doppler ultrasound, CT scan, and MRI is vital to prevent further complications [14].

Only a handful of OVT cases have been reported in COVID-positive patients. They imply the possibility of involvement of the ovarian vein as a site of CAC. Because of the spectrum of clinical features, along with a decline in reporting during the pandemic, there is a possibility that the actual magnitude of OVT cases is unknown. OVT should be kept in the differential while treating patients mimicking acute abdominal symptoms with a recent COVID-positive history. This extensive literature review aimed to analyze all available literature on OVT in patients with COVID-19 and emphasize its clinical importance in the differential diagnosis of acute abdominal symptoms in COVID-19 patients.

## Review

Methods

Identification of Research Question and Search Strategy

The research question for this literature review was "what is the association between ovarian vein thrombosis and COVID-19?" A systematic literature search was conducted using two electronic databases, PubMed and Google Scholar, to identify relevant articles published up to January 10, 2023. The following search terms were used: "ovarian vein thrombosis" OR "gonadal vein thrombosis" AND "2019 novel coronavirus disease" OR "COVID-19" OR "SARS-CoV-2 infection" OR "COVID-19 virus disease" OR "2019 novel coronavirus infection" OR "2019-nCoV infection" OR "coronavirus disease 2019" OR "coronavirus disease-19" OR "2019-nCoV disease" OR "COVID-19 virus infection." The search was supplemented by manual searching and retrieval of any additional articles that met the eligibility criteria.

Eligibility Criteria

Studies reporting cases of ovarian vein thrombosis in patients with COVID-19, studies published in English, studies published up to the search date, and studies in which the full-text article was available were included in this literature review.

Study Selection

Two independent reviewers screened the titles and abstracts of all retrieved articles to assess eligibility. Full-text articles were retrieved for all potentially eligible studies, and the same reviewers assessed them for inclusion. Any discrepancies were resolved through discussion.

Data Extraction and Quality Assessment

Data were extracted from the included studies by one reviewer using a standardized data extraction form. The extracted data included study design, patient demographics, clinical presentation, laboratory findings, imaging results, treatment, and outcome. The quality of the included studies was assessed by one reviewer using the Joanna Briggs Institute (JBI) Critical Appraisal Checklist for case reports and case series.

Data Synthesis and Reporting

A narrative synthesis of the findings was conducted, with a focus on the clinical characteristics, diagnosis, and management of ovarian vein thrombosis in patients with COVID-19. The Preferred Reporting Items for Systematic Reviews and Meta-Analyses (PRISMA) statement was used to report the methodology and findings of this literature review.

**Figure 1:**
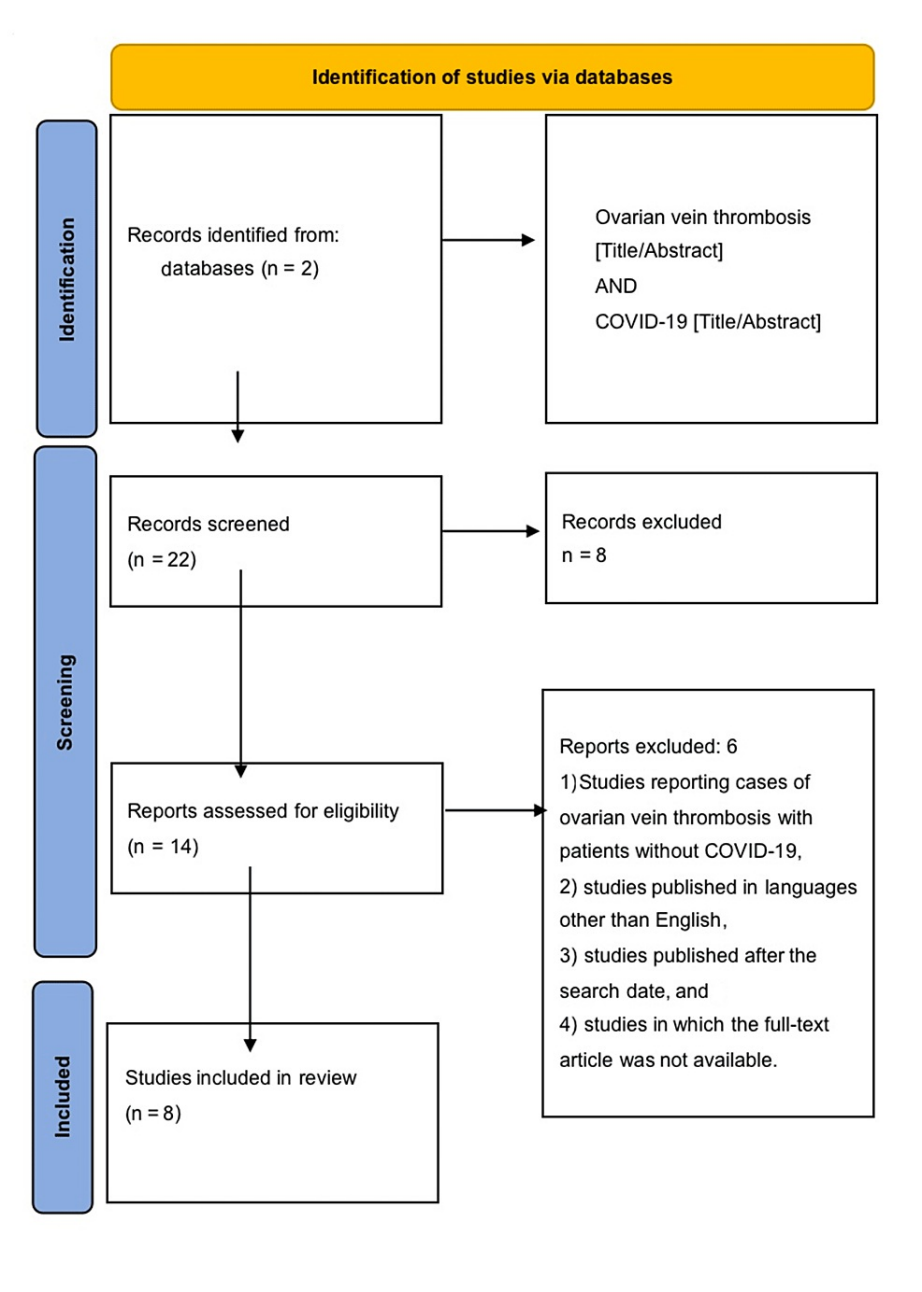
Flowchart of literature review search per Preferred Reporting Items for Systematic Reviews and Meta-analysis (PRISMA 2020) guidelines.

Ultimately, after a thorough screening of collected research, eight publications were included for extensive review. These included seven case reports and a case series of two cases. The PRISMA flowchart of the literature and search strategy of the studies is shown in Figure 1.

Results and discussion

Patient Demographics

After extensive analysis of the nine case reports available for ovarian vein thrombosis post-COVID-19, we found that almost all of the cases (77.7%) were in people older than 40 years. Only two cases, i.e, 22.22% were present in younger patients (<40 years). This validates the already established data for VTE being a diagnosis of older age [15,16]. There is an increase in the proportion of VTE cases with increasing age. A study done by Pasha et al. in 2021 noted a significant increase in VTE frequency with advancing age in COVID-positive population.

The association of ovarian vein thrombosis with pregnancy and postpartum is already established. Symptomatic postpartum OVT occurs in 0.01-0.05% of deliveries, especially in C-sections [17]. In this review, no significant obstetric history was found in the majority of the cases (77.77%) [6,11,13,14,18-20]. Pregnancy and post-partum were only associated with OVT in 22.22% of cases [14,21]. This points towards COVID as a possible etiological factor.

Pasha et al. observed in their study of 366 patients that most VTE events occurred within three weeks of a COVID-positive test [16]. It took about 40 days after COVID testing for VTE events to return to baseline. A 29-fold increase in VTE incidence was also reported during the first week post-COVID-positive detection. This was followed by a progressive decrease in the incidence of VTE until it reached basal values by the sixth week. Our review corroborates a similar finding. A total of 66.66% of patients presented within the first two weeks after a COVID-positive test [11,13,18-21]. The remaining 33.33% of patients took three to eight weeks for presentation [6,14].

Main comorbidities as described by various case reports include obesity (four cases), hypertension (three cases), and diabetes mellitus type 2 (two cases). These are also some of the most commonly reported characteristics in high-risk groups of VTE. Additionally, age over 40 years, history of a prior thrombosis, cardiac or respiratory failure, coronary artery disease, chronic liver or renal disease, cancer, autoimmune disease, etc. are some other associations of DVT [5,21,22]. These risk factors should be kept in mind while analyzing treatment response and severity of the disease. Demographic data extracted from the case reports can be seen in Table 1.

**Table 1:** Demographic data - (i) age and (ii) days from COVID-19-positive test to ovarian vein thrombosis diagnosis (OVT). OVT: ovarian vein thrombosis; COVID-19: coronavirus disease 2019

Case reports	Age	Days between COVID-positive detection and OVT diagnosis
Mohammadi et al. [21]	26	0
Veyseh et al. [13]	52	0
Fatimazahra et al. [14]	58	30
Fatimazahra et al. [14]	32	21
DeBoer et al. [18]	56	9
Vurture et al. [6]	46	48
Glanzer et al. [19]	56	11
Badrawi et al. [11]	41	0
Grewal et al. [20]	44	12

Clinical Profile

According to the available information, most thrombotic events in COVID patients occur in those critically ill, with severe pulmonary disease [13]. A study done by Klok et al. found 40.76% of cases of thrombosis out of 184 severe COVID-19 cases admitted to the ICU [23]. Another report stated that 60% of VTE cases are in ICU patients as opposed to 10% in general ward patients [24]. Contrary to this, our study finds evidence of COVID-associated pneumonia in only about 22% of cases of ovarian vein thrombosis [13,21].

Commonly, OVT presents as fever (80%) and right-sided pelvic pain (55%) [13]. Some may have a palpable cord-like abdominal mass [12]. Regardless of the underlying etiology, right ovarian vein is thrombosed in 70-90% of cases. This can be explained by compression of the right ovarian vein via dextroversion of the uterus, antegrade flow, and valvular incompetency of the right ovarian vein [13]. Our review identifies four patients with fever out of nine cases (44%) [6,11,14]. Out of those four, two patients present with concurrent chills, pointing towards septic pelvic thrombophlebitis [6,11]. Fever in septic pelvic thrombophlebitis is spiking and associated with chills [12]. Abdominal pain is reported in all cases of OVT post-COVID [6,11,13,14,18-21]. Clinical profile of the patients can be seen in Table 2.

**Table 2:** Spectrum of clinical symptoms in patients with ovarian vein thrombosis post-COVID-19. COVID-19: coronavirus disease 2019

Case report	Symptoms			
	Fever	Nausea/vomiting	Abdominal/flank pain	Anorexia
Mohammadi et al. [21]	Absent	Present	Present	Absent
Veyseh et al. [13]	Absent	Absent	Present	Absent
Fatimazahra et al. [14]	Present (low grade)	Present	Present	Present
Fatimazahra et al. [14]	Present (low grade)	Present	Present	Present
DeBoer et al. [18]	Absent	Absent	Present	Absent
Vurture et al. [6]	Present (with chills)	Present	Present	Absent
Glanzer et al. [19]	Absent	Present	Present	Absent
Badrawi et al. [11]	Present (with chills)	Present	Present	Absent
Grewal et al. [20]	Absent	Absent	Present	Absent

Although rare, OVT can cause life-endangering complications by extension of thrombus into systemic veins or pulmonary artery embolization. Two of the case reports suggest such an extension with one into iliac vein and the other into renal vein and pulmonary vein. Yet, it is unclear if these thrombi were a result of direct extension or primary response to hypercoagulable state. Nevertheless, these complications need to be watched out for as they can be fatal [13,21].

Laboratory Parameters

About 55.5% of OVT cases post-COVID have highly elevated D-dimer levels. A pooled analysis by Lippi and Favaloro in 2020 showcases increased elevations of D-dimer with increased severity of COVID and a worsening clinical picture [25]. Changes in prothrombotic factors like fibrinogen, D-dimer, factor VIII, and von Willebrand factor are responsible for the hypercoagulability associated with COVID-19. Additionally, ongoing inflammation associated with the presence of COVID-19 infection leads to rising levels of pro-inflammatory cytokines like C-reactive protein and ferritin. Such rise in acute phase reactants and pro-inflammatory cytokines is observed in many cases of OVT post-COVID. This further provides concrete evidence of the cross-link between inflammation and thrombosis [18,26]. Laboratory investigations of the cases included in the review are listed in Table 3.

**Table 3:** Laboratory parameters seen in various patients with ovarian vein thrombosis post-COVID-19. COVID-19: coronavirus disease 2019

Case report	Laboratory investigations		
	D-dimer (ng/mL)	CRP (mg/L)	Ferritin (ug/L)
Mohammadi et al. [21]	Normal	Normal	Normal
Veyseh et al. [13]	3813	217	719
Fatimazahra et al. [14]	1000	126	-
Fatimazahra et al. [14]	890	150	-
DeBoer et al. [24]	18,970	56.5	632
Vurture et al. [6]	Normal	Normal	Normal
Badrawi et al. [11]	2600	119.8	-

Diagnosis and Management

Prompt diagnosis and institution of early treatment modalities are crucial to reduce morbidity and mortality associated with OVT. Diagnosis of OVT can be done radiologically with ultrasound, magnetic resonance imaging (MRI) scan, or CT scan with IV contrast [27].

CT has been used as the modality of choice for diagnosing OVT. In our review, seven out of nine cases (78%) were diagnosed using a CT scan. A radiologic study has postulated that in the setting of OVT, CT is 100% sensitive and 99% specific. On the other hand, ultrasound is 50% sensitive and 99% specific, while MRI is 92% sensitive and 100% specific [28]. Positive CT findings show vein enlargement with a filling defect in the wall, and a central hypodensity representing the thrombus, or signs of perivascular edema [18,27]. One case reported the utilization of laparoscopy for the diagnosis of OVT [19].

The primary modality for diagnosis of OVT is Doppler ultrasound. However, CT and MRA confirm the diagnosis in case of uncertainty. Diagnosing ovarian vein thrombosis can be challenging because of the overlapping presentation with other differentials. A high index of suspicion should be kept in females presenting with abdominal pain. Standard guidelines for managing OVT and a systematic diagnostic protocol are unavailable [29].

Therapy of OVT varies from case to case and clinician to clinician. There is no official guideline for treatment. Bannow et al. suggest a short duration (three months) of anticoagulation, with the addition of antibiotics if an infection is suspected, in case of symptomatic OVT [17]. For asymptomatic cases, the use of anticoagulation is advised against, unless there is evidence of thrombus extension or pulmonary embolism. The only widely available treatment in CAC is a prophylactic dose of low molecular weight heparin like enoxaparin, which should be considered in all patients (including non-critically ill) who require hospital admission for COVID-19 infection [13]. While there is no particular regimen for treating VTE secondary to COVID-19 infection, the literature backs the use of apixaban or enoxaparin/acenocoumarol [6,11,13,14,18-21]. Diagnostic methods and management of the patients are stated in Table 4.

**Table 4:** Summary of studies depicting the diagnostic and treatment modalities utilized for OVT cases post-COVID-19. COVID-19: coronavirus disease 2019; OVT: ovarian vein thrombosis

Case report	Definitive diagnostic method utilized	Management	
		Short-term	Long-term (post-discharge)
Mohammadi et al. [21]	MRI	-	-
Veyseh et al. [13]	CT	Enoxaparin	Apixaban
Fatimazahra et al. [14]	CT	Enoxaparin	Acenocoumarol
Fatimazahra et al. [14]	CT	Enoxaparin	Acenocoumarol
DeBoer et al. [18]	CT	Apixaban	Apixaban
Vurture et al. [6]	CT	Enoxaparin + IV gentamycin + clindamycin	Amoxicillin/clavulanic acid + apixaban
Glanzer et al. [19]	Laparoscopy	Diagnostic laparoscopy + oophorectomy + heparin (post-op)	Apixaban
Badrawi et al. [11]	CT	Enoxaparin + cefuroxime + metronidazole	-
Grewal et al. [20]	CT	-	-

## Conclusions

COVID-associated coagulopathy is a rare but serious complication. It occurs almost as frequently as in other severe critical illnesses. COVID predisposes to a hypercoagulable state due to patient immobilization, adherence to ACE-2 receptors on endothelial cells, and venous stasis. Ovarian vein thrombosis in the context of COVID is infrequent but essential to know. OVT mimics the acute abdomen and has the potential of life-threatening complications if the diagnosis is missed. Hence, it should be kept on the differential for any woman with COVID positive history presenting with abdominal pain. Along with this, the presence of concurrent thromboses should be watched out for. CAC may be present even in the absence of respiratory symptoms and thus should not be ruled out until a definitive diagnosis is established. Laboratory investigations like D-dimer elevations may also provide a clue to diagnosing CAC but a definitive diagnosis requires CT scan or MRI. OVT is easily manageable if diagnosed and managed early.
